# Trends in maternal use of snus and smoking tobacco in pregnancy. A register study in southern Norway

**DOI:** 10.1186/s12884-019-2624-9

**Published:** 2019-12-16

**Authors:** Ellen Rygh, Frode Gallefoss, Liv Grøtvedt

**Affiliations:** 1 Research Department, Sørlandet Hospital, Kristiansand, Norway; 2 Chief of Clinical Research, Sørlandet Hospital, Kristiansand, Norway; 3 Faculty of Medicine, University of Bergen, Bergen, Norway; 4 Department Health and Inequality, Norwegian Institute of Public Health, Sandakerveien 24c, Bygg B, 0473 Oslo, Norway

**Keywords:** Maternal snus use, Maternal moist snuff use, Maternal cigarette smoking, Pregnancy, Tobacco, Quit rates

## Abstract

**Background:**

The use of tobacco products including Swedish snus (moist snuff) in pregnancy may cause adverse health outcomes. While smoking prevalence has decreased among fertile women in Norway, snus use has increased during the last years. We investigated whether these trends were reflected also during pregnancy in a population of women in Southern Norway.

**Methods:**

Data on smoking tobacco and snus use at three time points before and during pregnancy for 20,844 women were retrieved from the electronic birth record for the years 2012–2017. The results for the three-year period 2015–2017 were compared with a previously studied period 2012–2014. Prevalence and quit rates of tobacco use within groups of age, parity and education were reported. Within the same groups adjusted quit rates were analyzed using logistic regression. Mean birthweight and Apgar score of offspring were calculated for tobacco-users and non-users.

**Results:**

There was an increase of snus use before pregnancy from the period 2012–2014 to the period 2015–2017 from 5.1% (CI; 4.6 to 5.5) to 8.4% (CI; 7.8 to 8.9). Despite this, the use of snus during pregnancy did not increase from the first to the second period, but stabilized at 2.8% (CI; 2.5 to 3.2) in first trimester and 2.0% (CI; 1.7 to 2.2) in third trimester. Cigarette smoking decreased significantly both before and during pregnancy. Snus use and smoking during pregnancy were associated with a reduction in average birthweight, but no significant effects on Apgar scores. Odds ratios for quitting both snus and smoking tobacco during pregnancy were higher for women aged 25–34 years, for the primiparas and for those with a high level of education. Pregnant women were more likely to have quit tobacco use in the last time period compared to the first.

**Conclusions:**

While smoking during pregnancy was decreasing, the use of snus remained constant, levelling off to around 3% in first trimester and 2% in third trimester. Approximately 25% of those that used snus, and 40% that smoked before pregnancy, continued use to the third trimester. This calls for a continuous watch on the use of snus and other nicotine products during pregnancy.

## Background

The use of smoking tobacco has declined for decades in Norway. At the same time, the use of Swedish snus (moist snuff) has increased among young adults and especially in the last decade for women. In 2017, 15% of Norwegian women aged 16–44 years used snus daily or occasionally and likewise 14% used smoking tobacco [1, 2]. The health consequences from cigarette smoking in pregnancy are well known [1, 3–5]. Adverse health outcomes from use of snus during pregnancy are documented in recent years, especially from large population studies based on the Swedish Medical Birth Register. Snus use during pregnancy may increase risks of stillbirth, premature birth and small-for-gestational-age [3, 6–9], and is also associated with increased risk of oral cleft malformation and apnea in the newborn [4, 10, 11].

Due to long traditions of using and manufacturing snus, Sweden is granted a permanent exemption from the sales ban on snus in the European Union. Since Norway is not a member of the EU, snus is legally sold here too. Smoking in pregnancy has been reported in the Medical Birth Registry (MBR Norway) since 1999, and the prevalence has declined since the registration started [12]. National data on the use of snus in pregnancy is still not reported in the MBR Norway, although it has been registered in the electronic birth records (EBRs) and in the Health card for pregnant women (HCPW) Nationally since 2014. Thus, there is little knowledge on the extent of snus use in pregnancy in Norway. A register study from Southern Norway including the years 2012–2014 showed that the use of snus almost doubled during those three years, where 2% of women used snus and 7% smoked tobacco through pregnancy [13]. In a questionnaire study from Scandinavia, 6.5% used snus alone, and 4,8% used any other nicotine product at some time in pregnancy [14].

The aims of this study were to investigate self-reported tobacco use before and during pregnancy in the period 2015–2017, and to compare with the period 2012–2014 in the same region in Southern Norway [13], and further to identify potential predictors of tobacco use and cessation in sociodemographic groups. On the basis of the general trends in society [1, 2] and the previous study [13], our hypothesis was that there would be an increase in the proportion of snus use in pregnancy, but that a larger proportion of tobacco users would quit snus than cigarette smoking during pregnancy. We also compared birthweight and Apgar score in offspring of mothers who were users and non-users of snus or smoking tobacco in third trimester. As there is an ongoing shift from smoking tobacco to snus and newer nicotine products among young women of reproductive age in many countries, there is a need for more knowledge about the use in pregnancy and its consequences in clinical and preventive medicine.

## Methods

De-identified data were retrieved from the EBR for all women aged 16–44 years who gave birth in the years 2012–2017 at Sørlandet Hospital, comprising the maternity wards at three different hospitals located in the Agder counties in Southern Norway. The catchment area covers approximately 300,000 inhabitants. Almost all births in Norway take place in public institutions, and the majority in the local maternity ward.

The Norwegian Directorate of Health has drafted a standardized health record, “Health card for pregnant women” (HCPW) to be used in primary care. Health personnel interviewed the women about snus use and cigarette smoking at the first antenatal check-up and recorded this into the prescribed form as: “no”, “occasionally” or “daily”. This information was later transferred to the Hospital EBR as tobacco use “before pregnancy”. Tobacco use “in the first trimester” was registered into EBR by midwifes at the routine ultrasound check-up around week 18. Tobacco use “in the third trimester” was registered into EBR at the admission in the maternity ward. Since no objective measures confirmed quitting or abstinence, the information is referred to as self-reported. In this study we report daily and occasional tobacco use combined. We also report dual use of both snus use and smoking, which includes either a combination of both products occasionally, or one product daily and the other occasionally, or both products daily. Maternal age was grouped as: “16–24 years”, “25–34 years” and “35–44 years”. Parity was grouped as: “no previous birth”, “one previous birth” and “two or more previous births”. The self-reported highest achieved educational level at the time of pregnancy was recorded in the HCPW in three groups: “primary/lower secondary education” (less than high school), “upper secondary” (high school)” and “higher education” (university education or more than 4 years of college). Only women with complete data about tobacco use at all three registration points were included in the study population, and were followed from “before pregnancy” to “first trimester” and to “third trimester”.

The three variables age, parity and education from the main descriptive results of tobacco prevalence and quitting, were also considered as predictor variables in the adjusted analyses of quit rates. Age, parity and education have been indicated as possible determinants of smoking cessation in pregnancy [15, 16]. As the prevalence of snus use is rising while smoking is decreasing [12, 13], we found the possibility to study also the tendency to quit tobacco in a time perspective important. Thus, we included the birth year of the newborn in the adjusted analyses.

### Statistical analysis

Data retrieval was undertaken by Sykehuspartner (the Hospital’s ICT Trust), transferred to the Norwegian Institute of Public Health, and analyzed with STATA (version 15).

The three-year-periods 2015–2017 and 2012–2014 were analyzed separately and compared. The use of snus and smoking tobacco before pregnancy, in the first and third trimester were presented as prevalence (95% CI) in groups of age, parity, and education. The proportions of switching from snus to cigarettes, or opposite, were also calculated.

The quit rates (95% CI) were calculated as the proportion of women who reported tobacco use before pregnancy (N1), but no tobacco use in the first trimester (N2) or no tobacco use in the third trimester (N3): Quit rates from before pregnancy to first trimester: N1-N2/N1*100. Quit rates from before pregnancy to third trimester: N1-N3/N1*100. In addition to presenting prevalence and quit rates for the women that were dual users of both snus and smoking tobacco, we analyzed the proportion that quit both products or that continued just one of the products.

Logistic regression was applied in the multivariable analyses for quit rates. The study population for the whole period 2012–2017 was included in separate analyses for snus use and smoking tobacco. The outcome variable for quitting snus use or not was: 0) “continued snus use in third trimester” and 1) “no snus use in third trimester”. The outcome variable for quitting smoking or not was: 0) “continued smoking in third trimester” and 1) “no smoking in third trimester”. These 0/1 outcome variables corresponded to the quit rates among women who reported tobacco use before pregnancy, but no tobacco use in the third trimester. The outcome measures “Odds Ratios” (OR) were given with confidence intervals (95% CI) and *P*-values. In the regression analyses, the predictor variables age, parity and education were all categorized with three levels as explained above in the previous section. Time period, based on birth year of the newborn, was included as predictor-variable with the years 2015–2017 versus the years 2012–2014. In addition to the logistic regression, quit rates in percent were calculated for the entire period 2012–2017, representing the unadjusted values of tobacco quitting.

For the period 2015–2017 mean birth weights with confidence intervals and *P*-values (95% CI) for offspring of snus users and smokers were compared to mean birth weight for offspring of the non-tobacco users in third trimester. Here, all women in the age 16–44 years with records of snus use or cigarette smoking in the third trimester were included in the calculations, not only those with complete records of both snus use and smoking at all three time points. Newborns with birth weight below 500 g were not included in the analyses (unviable births). Corresponding analyses with mean values (95% CI) were performed for Apgar score.

## Results

In the period 2012–2017 there were 20,844 births at Sørlandet hospital, comprising 5.9% of all births in Norway in these years. Missing values for snus use before pregnancy, in first and third trimester were 1.2, 2.2 and 3.2%, respectively. Correspondingly, missing values for smoking tobacco were 1.1, 0.5 and 2.5%; for Apgar score 1 min and 5 min 0.3% for both time points, and for Apgar score 10 min 1.4%. For birthweight, missing information constituted 0.3%, including omitted births with infant weight below 500 g (*N* = 58). Missing information on education was 6.9%; 8.6% in the age group 16–24 years, 5.8% in the age group 25–34 years and 7.7% in the age group 35–44 years.

Women below age 16 (*n* = 2) and above age 44 (*n* = 40), were excluded from the study population. The study population cohort constituted 19,767 women (95% of all 20,844) who had complete reports of tobacco use, 9912 in the period 2012–2014 and 9855 in 2015–2017.

### Time trends

**Fig. 1:**
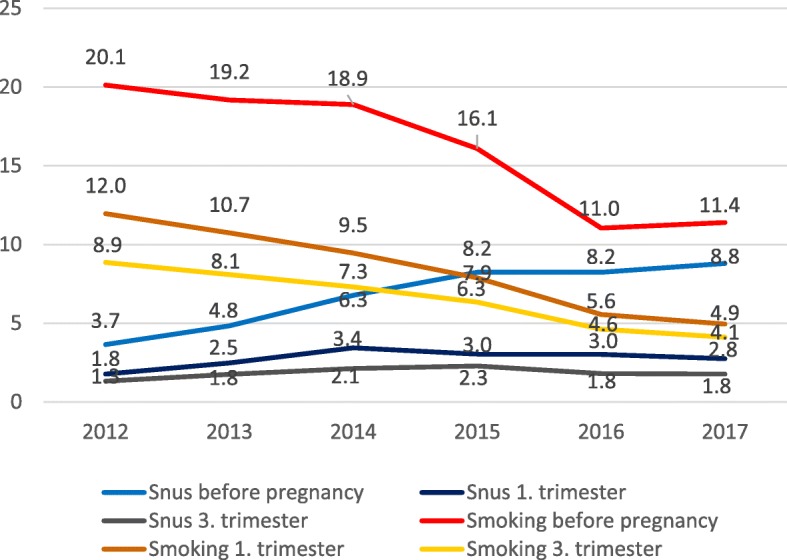
Time trends in snus use and cigarette smoking among pregnant women 2012–2017. Percent

Time trends for the prevalence of pregnancy tobacco use comprising the six years 2012–2017 are visualized in Fig. 1. Further, time trends comparing the three-year periods 2015–2017 and 2012–2014 are presented in Table 1 and Additional file 1: Table S1. Comparing the two three-year periods, there was an increase in the self-reported snus use before pregnancy from 5.1% (CI; 4.6 to 5.5) to 8.4% (CI 7.8 to 8.9). Despite this, use of snus during pregnancy in the last period remained unchanged compared to the first period, with 2.8% in the first trimester and 2.0% in the third trimester. For cigarette smoking there was a reduction before pregnancy from 19.2% (CI; 18.5 to 20.0) in the first period to 12.8% (CI; 12.1 to 13.5%) in the last period. Correspondingly, in the third trimester smoking was reduced from 8.1% (CI; 7.6 to 8.7) to 5.0% (CI; 4.6 to 5.5) (Table 1, Additional file 1: Table S1).

**Table 1:** Snus use and cigarette smoking among pregnant women 2015–2017.^a^ Age 16–44 years. Percent, 95% Cl. *N* = 9855

	Before pregnancy			First trimester			Third trimester		
	N	%	CI	N	%	CI	N	%	CI
Snus use, all									
Snus use, occasional (*n* = 9855)	159	1.6	1.4–1.9	108	1.1	0.9–1.3	72	0.7	0.6–0.9
Snus use, daily (*n* = 9855)	665	6.7	6.3–7.3	169	1.7	1.5–2.0	120	1.2	1.0–1.5
Snus use, daily and occasional (*n* = 9855)	824	8.4	7.8–8.9	277	2.8	2.5–3.2	192	2.0	1.7–2.2
Snus use in age groups									
16–24 years (*n* = 1285)	264	20.5	18.4–22.9	103	8.0	6.6–9.6	75	5.8	4.6–7.3
25–34 years (*n* = 6660)	500	7.5	6.9–8.2	153	2.3	2.0–2.7	104	1.6	1.3–1.9
35–44 years (*n* = 1910)	60	3.1	2.4–4.0	21	1.1	0.7–1.7	13	0.7	0.4–1.2
Snus use in groups of parity									
No previous child (*n* = 3922)	512	13.1	12.0–14.1	144	3.7	3.1–4.3	96	2.4	2.0–3.0
One previous child (*n* = 3570)	226	6.3	5.6–7.2	93	2.6	2.1–3.2	65	1.8	1.4–2.3
Two or more previous children (*n* = 2363)	86	3.6	2.9–4.5	40	1.7	1.2–2.3	31	1.3	0.9–1.9
Snus use in educational groups ^b^									
Primary/lower secondary (*n* = 734)	60	8.2	6.3–10.4	36	4.9	3.5–6.7	25	3.4	2.2–5.0
Upper secondary (*n* = 3541)	412	11.6	10.6–12.7	161	4.5	3.9–5.3	125	3.5	2.9–4.2
Higher education (*n* = 4987)	314	6.3	5.6–7.0	63	1.3	1.0–1.6	32	0.6	0.4–0.9
Smoking, all									
Smoking, occasional (*n* = 9855)	285	2.9	2.6–3.2	101	1.0	0.8–1.2	90	0.9	0.7–1.1
Smoking, daily (*n* = 9855)	974	9.9	9.3–10.5	496	5.0	4.6–5.5	407	4.1	3.7–4.5
Smoking, daily or occasional (*n* = 9855)	1259	12.8	12.1–13.5	597	6.1	5.6–6.5	497	5.0	4.6–5.5
Smoking in age groups									
16–24 years (*n* = 1285)	272	21.2	19.0–23.5	136	10.6	9.0–12.4	109	8.5	7.0–10.1
25–34 years (*n* = 6660)	780	11.7	10.9–12.5	351	5.3	4.7–5.8	294	4.4	3.9–4.9
35–44 years (*n* = 1910)	207	10.8	9.5–12.3	110	5.8	4.8–6.9	94	4.9	4.0–6.0
Smoking in groups of parity									
No previous child (*n* = 3922)	570	14.5	13.4–15.7	227	5.8	5.1–6.6	184	4,7	4.1–5.4
One previous child (*n* = 3570)	409	11.5	10.4–12.5	203	5.7	4.9–6.5	170	4,8	4.1–5.5
Two or more previous children (*n* = 2363)	280	11.9	10.6–13.2	167	7.1	6.1–8.2	143	6,1	5.1–7.1
Smoking in educational groups ^b^									
Primary/lower secondary (*n* = 734)	187	25.5	22.4–28.8	124	16.9	14.3–19.8	108	14.7	12.2–17.5
Upper secondary (*n* = 3541)	713	20.1	18.0–21.5	348	9.8	8.9–10.9	296	8.4	7.5–9.3
Higher education (*n* = 4987)	275	5.5	4.9–6.2	75	1.5	1.2–1.9	59	1.2	0.9–1.5
Dual use, all	142	1.4	1.2–1.7	33	0.3	0.2–0.5	13	0.1	0.1–2.3
Dual use in age groups									
16–24 years (*n* = 1285)	61	4.8	3.7–6.1	14	1.1	0.6–1.8	2	0.2	0.0–0.6
25–34 years (*n* = 6660)	72	1.1	0.8–1.4	18	1.1	0.2–0.4	9	0.1	0.1–0.3
35–44 years (*n* = 1910)	9	0.5	0.2–0.9	1	1.1	0.0–0.3	2	0,1	0.0–0.4

^a^Tobacco use: daily and occasional use combined

^b^In comparing the levels of completed education, the age 25+ is often used in official statistics in Norway, as most people then are considered to have completed their education. As pregnancy may prevent or delay the completion of education, we included all ages in the calculations of educational level in this study. This may also give a truer picture of the predominantly young snus users

### Pregnancy tobacco use in the period 2015–2017

In the youngest age group 16–24 years 20.5% used snus before pregnancy, while the corresponding prevalence in the age group 25–34 years was 7.5%. (Table 1). Likewise, 21.2% of the youngest smoked before pregnancy, while the corresponding prevalence for the 25–34 year group was 11.7% (Table 1). Only 1.4% of the women reported dual use of both snus and smoking tobacco before pregnancy, and the proportion was below 1% during pregnancy. Dual use was most marked among the youngest aged 16–24 years, approximating to 5% before pregnancy (Table 1).

While the prevalence of snus use before pregnancy was highest in women with upper secondary education, cigarette smoking was most prevalent among women with primary/lower secondary education. No difference in snus use during pregnancy (first and third trimester) was found between women with primary and secondary education. Women with higher education had the lowest prevalence of snus use at all three time points. The educational differences in smoking prevalence were pronounced at all three time points before and during pregnancy. Around 6% of women with higher education were tobacco users prior to pregnancy (snus and/or smoke), but the prevalence was only 1–2% during pregnancy (Table 1).

### Quit rates

Out of 824 women who had used snus prior to pregnancy in the period 2015–2017, 66.4% had quit by the first trimester and 76.7% by the third trimester. Out of 1259 who smoked before pregnancy, 52.6% had quit by the first trimester and 60.5% by the third trimester. Thus, a larger proportion had quit snus than smoking tobacco during pregnancy (Table 2).

**Table 2:** Quit rates for pregnancy snus use and cigarette smoking 2015–2017.^a^ Age 16–44 years. Percent, 95% Cl. *N* = 9855.

	From before pregnancy to first trimester		From before pregnancy to third trimester	
	%	CI	%	CI
Snus use, all 16–44 years (*N* = 824 before pregnancy)	66.4	63–70	76.7	74–80
Snus use, age groups				
16–24 years	61.0	55–67	71.6	66–77
25–34 years	69.4	65–73	79.2	75–83
35–44 years	65.0	52–77	78.3	66–88
Snus use, groups of parity				
No previous child	71.9	68–76	81.3	78–85
One previous child	58.8	52–65	71.2	65–77
Two or more previous children	53.5	42–64	64.0	53–74
Snus use, educational groups				
Primary/lower secondary	40.0	28–53	58.3	45–71
Upper secondary	60.9	56–66	69.7	65–74
Higher education	79.9	75–84	89.8	86–93
Smoking, all 16–44 years (*N* = 1259 before pregnancy)	52.6	50–55	60.5	58–63
Smoking, age groups				
16–24 years	50.0	44–56	59.9	54–66
25–34 years	55.0	51–59	62.3	59–66
35–44 years	46.9	40–54	54.6	48–62
Smoking, groups of parity				
No previous child	60.2	56–64	67.7	64–72
One previous child	50.4	45–66	58.4	53–63
Two or more previous children	40.4	35–46	48.9	43–55
Smoking, educational groups				
Primary/lower secondary	33.7	27–41	42.2	35–50
Upper secondary	51.2	47–55	58.5	55–62
Higher education	72.7	67–78	78.5	73–83
Dual use, all 16–44 years ^b^ (*N* = 142 before pregnancy)	76.8	69–83	90.8	85–95

^a^Tobacco use: daily and occasional use combined

^b^The quit rates for dual use include those who quit both products, as well as those who quit only one of the products. See text description

Compared to the previous three-year period 2012–2014 (Additional file 2: Table S2), a larger proportion had quit snus by first trimester in the period 2015–2017: 66.4% vs 51.3%. Correspondingly, in the third trimester the figures were 76.7% vs 66.1%, respectively. The proportions that quit smoking by first trimester were 52.6% in the last period compared to 44.9% in the first period. Corresponding figures for the third trimester were 60.5% vs 57.7%, respectively. Hence, the bivariate analyses showed higher quit rates for snus use during pregnancy in the last period compared to the first, while the same was not true for smoking by the third trimester (Table 2, Additional file 2: Table S2).

The quit rates for dual use were 76.8% from before pregnancy to first trimester, and 90.8% from before pregnancy to third trimester (Table 2). However, the dual users partly switched to use only one tobacco product. When taking this into account, 46.5% of the dual users had quit all tobacco by first trimester and 60.6% by third trimester. We also found that 3.6% of the women who had used snus before pregnancy had switched from snus use to smoking by third trimester. Correspondingly, 2.2% had switched from smoking to snus use by third trimester (data not shown). Lower quit rates, but generally the same pattern of switching among the dual users were found in the first period (59.1% by first trimester and 81.1% by third trimester, see Additional file 2: Table S2).

Table 3 shows unadjusted quit rates in percent and adjusted ORs for quitting snus use and smoking during pregnancy for the whole period 2012–2017. Women aged 25–34 years had higher ORs for quitting both snus and smoking tobacco than women aged 16–24 years. Women with no previous children (parity 0) were more likely to quit snus and smoking tobacco than those with previous births. Women with higher education were most likely to quit snus or smoking during pregnancy, however more so for smoking (OR > 5) than for snus use (OR > 4). Pregnant women were more likely to have quit tobacco use during pregnancy in the last period (2015–2017), compared to the first (2012–2014).

**Table 3:** Quit rates^a^ for tobacco use during pregnancy 2012–2017, expressed as percent (unadjusted) and OR (adjusted)

	Snus users (*n* = 1325 before pregnancy)				Smokers (*n* = 3165 before pregnancy)			
	%	OR	95% CI	P-value	%	OR	95% CI	P-value
Age groups								
16–24 years	67.1	1	Ref	–	54.4	1	Ref	–
25–34 years	75.7	1.38	1.00–1.89	0.051	61.2	1.29	1.06–1.58	0.011
35–44 years	75.3	1.69	0.84–3.42	0.142	56.8	1.15	0.86–1.54	0.331
Parity								
Two or more previous children	61.0	1	Ref	–	45.9	1	Ref	–
One previous child	66,8	1.02	0.62–1.68	0.941	56.7	1.56	1.25–1.94	0.000
No previous children	76.9	2.00	1.22–3.28	0.006	66.0	2.55	2.04–3.19	0.000
Education								
Primary, lower secondary	57.0	1	Ref	–	42.6	1	Ref	–
Upper secondary	64.5	1.30	0.83–2.03	0.248	55.3	1.64	1.33–2.02	0.000
Higher education	86.8	4.34	2.57–7.33	0.000	80.0	5.18	3.94–6.83	0.000
Time period								
2012–2014	66.1	1	Ref	–	57.7	1	Ref	–
2015–2017	76.7	1.70	1.28–2.24	0.000	60.5	1.18	1.00–1.38	0.044

Note: Time period with all six years as a continuous variable gave minimal changes to the ORs compared to the dichotomous variable in the model above. Only predictors found to alter the ORs were included in the main analyses. Interaction between the predictors parity and mothers age was tested, but was not found to be statistically significant

^a^Women who reported tobacco use before pregnancy, but no tobacco use in third trimester

### Birth weight

The study population for analyzing differences in birth weight for the period 2015–2017 was 102,011 women. The average birthweight for children of tobacco-free mothers in the third trimester (*n* = 9213) was 3524 g (CI; 3513 to 3535). The average birthweight for children of mothers who had been daily or occasional smokers during third trimester (*n* = 506) was 3278 g (CI; 3229 to 3328, *P*-value <0.001), thus a statistically significant reduction of 246 g in birthweight. Average birthweight for children of mothers who had used snus daily or occasionally in the third trimester (*n* = 201) was 3418 g (CI; 3338 to 3498, *P*-value <0.006 ), thus a smaller, but also a statistically significant reduction in average birthweight of 106 g. We studied the distribution of birth weight in groups of maternal age, parity and education. Significant lower birth weight was found in children of smoking mothers than in the non-tobacco users in all three age groups, as well as in all groups of parity and education (Additional file 3). The difference in birth weight between children of snus users and non-tobacco users was statistically significant among mothers aged 16–24 years and among those with upper secondary education. Snus users with parity 0 contributed more than the other groups to the reduced birth weight (Additional file 3).

There were no statistically or clinically significant differences in Apgar scores in children of mothers who had used snus or smoked cigarettes during pregnancy compared to the non-tobacco users.

## Discussion

This study on self-reported tobacco use showed that the proportion that used snus *before* pregnancy more than doubled from 2012 until 2017. The proportion that used snus *during* pregnancy increased moderately in the years 2012–2014, but then remained quite constant from 2015 to 2017. This implies that the increase in snus use in the general population in the period 2015–2017 was not mirrored by a corresponding increase during pregnancy. The use of smoking tobacco declined both before pregnancy and during pregnancy in the same period. A larger proportion of tobacco users quit snus than smoking tobacco during pregnancy in both time periods.

The strength of this study lies in the large study population, with complete information about tobacco use at three time points before and during pregnancy from the participating cohort of women. Another strength is the low rates of missing information. It would be a further strength if the study had been nationwide, but National reporting of snus use in pregnancy is still not implemented in Norway. This fact implies a greater demand for regional data on snus use during pregnancy. An objective measure of quitting/abstinence from snus or smoking (cotinine) would also have increased the validity and the general applicability of our results. At the national level, the use of snus among non-pregnant women was higher than the observed prevalence before pregnancy in our study. Also, the prevalence of smoking at the beginning of the first trimester was lower for Norway as a whole than for our study population in Southern Norway [17]. One explanation may be that women in this region traditionally have had lower prevalence of snus use and higher use of smoking tobacco than the national averages [1, 2]. There could also have been an under-reporting in our study because of the stigma associated with tobacco use in pregnancy. Gunnerbeck et al. found that the Swedish MBR misclassified almost 45% of the cotinine-verified users of snus as non-users in late pregnancy [18], while Mattsson et al. found a high agreement between cotinine levels and smoking data in the Swedish MBR [19]. Whether there is a possible, systematic underreporting of tobacco use in the MBRs is a task for further research. Finally, we may assume that the lower snus prevalence seen in this study partly indicate that young women quit or restrain from tobacco products not only during pregnancy, but also prior to pregnancy, and especially in the period when they plan to get pregnant [20].

There has been increased focus in recent years on the harmful effects of snus in pregnancy. In spring 2016, the Norwegian Directorate for Health launched a media campaign against snus use in pregnancy, which was cited nationwide in newspapers as well as on the web and television. We have scarce information whether snus use has become a topic in antenatal care in Norway. But revision of routines after 2014, where the HCPW now also calls for registration of snus use from the first antenatal check-up, may also have contributed to augmented awareness, both among the women and among health personnel.

Snus use among Norwegian non-pregnant women in fertile age was approximately the double of that in Sweden [2, 21]. In our study population of pregnant women in Southern Norway the prevalence of snus use in the first trimester was about threefold of that in Sweden: 3,0% versus 1,2% in 2016 [22]. This indicates that snus use among women has become a larger problem in Norway than in Sweden. The prevalence of smoking in Norway and Sweden was comparable, approximately 15% among non-pregnant women 16–44 years in 2016, and in the first trimester around 5% in both countries [17].

The reduction in birthweight by snus use and smoking tobacco in this study was not adjusted for potential confounders like gestational age, since our dataset did not include information on this. The observed difference in birth weight between snus users and non-tobacco users in our study population seem mainly to be influenced by women in the youngest age group and those with medium education and no previous child. The association of birth weight reduction by snus use in pregnancy is debated, and recent studies indicate that smokeless tobacco use has minor effects on birth weight [23, 24]. The lack of difference in Apgar score between the cigarette smokers/snus users and the tobacco-free groups is to our knowledge not reported previously.

Cigarette smoking and snus use before and during pregnancy was most prevalent at young age and lower educational level. But snus use before and in pregnancy was also prevalent among women with intermediate education. This is consistent with findings among non-pregnant women in Norway [25]. Smokers with primary and upper secondary education constituted the bulk of tobacco use, both before and during pregnancy. Overall, 77% of the women using snus and 61% of the cigarette smokers had stopped by the third trimester. In a recent study from Scandinavia (Oslo and Stockholm), the quit rates were even higher, possibly because here the participants mainly were recruited from urban populations [14]. In our study, higher education was a powerful predictor of quitting, regarding both snus use and smoking. The same was true for women delivering their first baby compared to those of previous parity. This was in accordance with findings in a study about smoking in pregnancy from the US [16].

We found low prevalence for dual use in the studied population compared to studies among non-pregnant women [25, 26]. A comparable low prevalence of dual use in pregnancy was found in the Scandinavian study [14]. Among the women reporting dual use before pregnancy in our study, there was a shift towards a higher proportion being “smokers only” compared to “snus users only” in the third trimester from the first three-year period to the second. This may be a result of the increased focus of the harmful effects of snus as mentioned above.

Although the proportion that quit snus and smoking tobacco increased in the last 3-year period, still a considerable proportion of women who smoked or used snus before pregnancy continued through pregnancy. A portion of snus, as it is sustained longer in the mouth, may result in a higher and longer-lasting concentration of nicotine in the blood than a smoked cigarette. Nicotine quickly passes through the placenta barrier, and in one study nicotine concentration was 15% higher in the fetal than in the maternal plasma [27]. Perinatal snus exposure has shown long-term effect on cardiovascular function in the child [28]. Nicotine exposure in pregnancy may also have adverse effects on fetal brain and lung development [29, 30]. Since no lower threshold of harmful effect of nicotine on the fetus or child is known, this represents a potential public health challenge [31]. In North America there has been an increase in use of moist snuff and other nicotine delivery products [32]. Advice on the harmful effects of all nicotine products should therefore be provided to adolescent girls. A possible arena for information may be in the context of consultations on contraception.

Assuming that the trends in the period 2015–2017 continue, our study may give a small indication that Norway might be heading towards tobacco-free pregnancies. However, we are far from there yet. Snus use is a relatively recent trend among women in Norway. Many have started at an early age and are not concurrent smokers [25]. Snus and new nicotine products like e-cigarettes are advocated as harm reduction products to cigarette smokers [33]. The Scandinavian countries, especially Sweden and Norway, have been a niche for the sale of snus. The tobacco industry is fighting to open legal sales in the rest of the EU. Unfortunately, often when snus is promoted as a less harmful substitute for smoked tobacco, the potential harmful effects of nicotine exposure in pregnancy seem clearly under-communicated [34].

## Conclusion

While smoking decreased, the use of snus during pregnancy remained constant in this study. The quit rates for snus were higher than for smoking tobacco. The OR for quitting both snus and cigarettes were higher for women aged 25–34 years, for woman delivering their first baby and for those with higher education. Around one in four of the snus users and two of five of the smokers continued through pregnancy, which implies a need for surveillance and preventive measures regarding the use of snus and other nicotine products during pregnancy.

## Supplementary information

**Additional file 1: Table S1.** Pregnancy snus use and cigarette smoking 2012–2014. Age 16–44 years. Percent. 95% Cl. *N* = 9912.

**Additional file 2: Table S2.** Quit rates for pregnancy snus use and cigarette smoking 2012–2014. Age 16–44 years. Percent. 95% Cl. N = 9912.

**Additional file 3.** Stratification of birth weight among snus users, smokers and non-tobacco users.

## Data Availability

The data that support the findings of this study are available from Sykehuspartner (the Hospital’s ICT Trust at Sørlandet Hospital), but restrictions apply to the availability of these data, which were used under license for the current study, and are therefore not publicly available. Data are however available from the authors upon reasonable request and with permission of the Regional Committee on Medical and Health Research Ethics.

## Abbreviations

EBR: Electronic Birth Record
HCPW: Health Card for Pregnant Women
MBR: Medical Birth Registry
