# Supplementary material for: Trends in maternal use of snus and smoking tobacco in pregnancy. A register study in southern Norway
Source: BMC Pregnancy Childbirth. 2019 Dec 16;19:500. doi: 10.1186/s12884-019-2624-9 (PMC6915947; doi:10.1186/s12884-019-2624-9)
Supplement: Supplementary file 1 — Additional file 1: Table S1. Pregnancy snus use and cigarette smoking 2012–2014. Age 16–44 years. Percent. 95% Cl. N = 9912. [file 12884_2019_2624_MOESM1_ESM.docx]

**Table S1. Pregnancy snus use and cigarette smoking 2012-2014.* Age 16-44 years. Percent. 95% Cl. N=9912**

|  | Before pregnancy | | | First trimester | | | Third trimester | | |
| --- | --- | --- | --- | --- | --- | --- | --- | --- | --- |
|  | N | % | CI | N | % | CI | N | % | CI |
| **Snus use, all** |  |  |  |  |  |  |  |  |  |
| Snus use, occasional (n=9912) | 166 | 1.7 | 1.4-1.9 | 111 | 1.1 | 0.9-1.3 | 82 | 0.8 | 0.7-1.0 |
| Snus use, daily (n=9912) | 335 | 3.4 | 3.0-3.8 | 133 | 1.3 | 1.1-1.6 | 88 | 0.9 | 0.7-1.1 |
| Snus use, daily and occasional (n=9912) | 501 | 5.1 | 4.6-5.5 | 244 | 2.5 | 2.2-2.8 | 170 | 1.7 | 1.5-2.0 |
|  |  |  |  |  |  |  |  |  |  |
| **Snus use in age groups** |  |  |  |  |  |  |  |  |  |
| 16-24 years (n=1581) | 195 | 12.3 | 10.8-14.1 | 111 | 7.0 | 5.8-8.4 | 76 | 4.8 | 3.8-6.0 |
| 25-34 years (n=6590) | 273 | 4.1 | 3.7-4.7 | 121 | 1.8 | 1.5-2.2 | 84 | 1.3 | 1.0-1.6 |
| 35-44 years (n=1741) | 33 | 1.9 | 1.3-2.7 | 12 | 0.7 | 0.4-1.2 | 10 | 0.6 | 0.3-1.1 |
|  |  |  |  |  |  |  |  |  |  |
| **Snus use in groups of parity** |  |  |  |  |  |  |  |  |  |
| No previous child (n=3962) | 335 | 8.5 | 7.6-9.4 | 154 | 3.9 | 3.3-4.5 | 100 | 2.5 | 2.1-3.1 |
| One previous child (n=3678) | 129 | 3.5 | 2.9-4.2 | 65 | 1.8 | 1.4-2.2 | 53 | 1.4 | 1.1-1.9 |
| Two or more previous children (n=2272) | 37 | 1.6 | 1.1-2.2 | 25 | 1.1 | 0.7-1.6 | 17 | 0.7 | 0.4-1.2 |
|  |  |  |  |  |  |  |  |  |  |
| **Snus use in educational groups **** |  |  |  |  |  |  |  |  |  |
| Primary/lower secondary (n=779) | 40 | 5.1 | 3.7-6.9 | 28 | 3.6 | 2.4-5.2 | 18 | 2.3 | 1.4-3.6 |
| Upper secondary (n=3623) | 247 | 6.8 | 6.0-7.7 | 145 | 4.0 | 3.4-4.7 | 109 | 3.0 | 2.5-3.6 |
| Higher education (n=4802) | 186 | 3.9 | 3.3-4.5 | 54 | 1.1 | 0.8-1.5 | 34 | 0.7 | 0.5-1.0 |
|  |  |  |  |  |  |  |  |  |  |
| **Smoking, all** |  |  |  |  |  |  |  |  |  |
| Smoking, occasional (n=9912) | 415 | 4.2 | 3.8-4.6 | 173 | 1.7 | 1.5-2.0 | 126 | 1.3 | 1.1-1.5 |
| Smoking, daily (n=9912) | 1491 | 15.0 | 14.3-15.8 | 877 | 8.8 | 8.3-9.4 | 680 | 6.9 | 6.4-7.4 |
| Smoking, daily or occasional (n=9912) | 1906 | 19.2 | 18.5-20.0 | 1050 | 10.6 | 10.0-11.2 | 806 | 8.1 | 7.6-8.7 |
|  |  |  |  |  |  |  |  |  |  |
| **Smoking in age groups** |  |  |  |  |  |  |  |  |  |
| 16-24 years (n=1581) | 526 | 33.3 | 30.9-35.7 | 350 | 22.1 | 20.1-24.3 | 255 | 16.1 | 14.3-18.0 |
| 25-34 years (n=6590) | 1131 | 17.2 | 16.3-18.1 | 577 | 8.8 | 8.1-9.5 | 448 | 6.8 | 6.2-7.4 |
| 35-44 years (n=1741) | 249 | 14.3 | 12.7-16.0 | 123 | 7.1 | 5.9-8.4 | 103 | 5.9 | 4.9-7.1 |
|  |  |  |  |  |  |  |  |  |  |
| **Smoking in groups of parity** |  |  |  |  |  |  |  |  |  |
| No previous child (n=3962) | 928 | 23.4 | 22.1-24.8 | 460 | 11.6 | 10.6-12.6 | 325 | 8.2 | 7.4-9.1 |
| One previous child (n=3678) | 593 | 16.1 | 14.9-17.4 | 340 | 9.2 | 8.3-10.2 | 264 | 7.2 | 6.4-8.1 |
| Two or more previous children (n=2272) | 385 | 16.9 | 15.4-18.6 | 250 | 11.0 | 9.7-12.4 | 217 | 9.6 | 8.4-10.8 |
|  |  |  |  |  |  |  |  |  |  |
| **Smoking in educational groups **** |  |  |  |  |  |  |  |  |  |
| Primary/lower secondary n=779) | 280 | 35.9 | 32.6-39.4 | 203 | 26.1 | 23.0-29.3 | 160 | 20.5 | 17.8-23.5 |
| Upper secondary (n=3623) | 1040 | 28.7 | 27.2-30.2 | 633 | 17.5 | 16.2-18.7 | 487 | 13.4 | 12.3-14.6 |
| Higher education (n=4802) | 446 | 9.3 | 8.5-10.1 | 124 | 2.6 | 2.2-3.1 | 85 | 1.8 | 1.4-2.2 |
|  |  |  |  |  |  |  |  |  |  |
| **Dual use, all** | 132 | 1.3 | 1.1-1.6 | 54 | 0.5 | 0.4-0.7 | 25 | 0.3 | 0.2-0.4 |
|  |  |  |  |  |  |  |  |  |  |
| **Dual use in age groups** |  |  |  |  |  |  |  |  |  |
| 16-24 years (n=1581) | 60 | 3.8 | 2.9-4.9 | 35 | 2.2 | 1.5-3.1 | 14 | 0.9 | 0.5-1.5 |
| 25-34 years (n=6590) | 67 | 1.0 | 0.8-1.3 | 17 | 0.3 | 0.2-0.4 | 9 | 0.1 | 0.1-0.3 |
| 35-44 years (n=1741) | 5 | 0.3 | 0.1-0.7 | 2 | 0.1 | 0.0-4.1 | 2 | 0.1 | 0.0-0.4 |

***** Tobacco use: daily and occasional use combined

****** In comparing the levels of completed education, the age 25+ is often used in official statistics in Norway, as most people then are considered to have completed their education. As pregnancy may prevent or delay the completion of education, we included all ages in the calculations of educational level in this study. This may also give a truer picture of the predominantly young snus users.
