# Supplementary material for: Trends in maternal use of snus and smoking tobacco in pregnancy. A register study in southern Norway
Source: BMC Pregnancy Childbirth. 2019 Dec 16;19:500. doi: 10.1186/s12884-019-2624-9 (PMC6915947; doi:10.1186/s12884-019-2624-9)
Supplement: Supplementary file 2 — Additional file 2: Table S2. Quit rates for pregnancy snus use and cigarette smoking 2012–2014. Age 16–44 years. Percent. 95% Cl. N = 9912. [file 12884_2019_2624_MOESM2_ESM.docx]

**Table S2. Quit rates for pregnancy snus use and cigarette smoking 2012-2014.* Age 16-44 years. Percent. 95% Cl. N=9912**

|  | **From before pregnancy to first trimester** | | **From before pregnancy to third trimester** | |
| --- | --- | --- | --- | --- |
|  | % | CI | % | CI |
| **Snus use, all**  (N=501 before pregnancy) | 51.3 | 47-56 | 66.1 | 62-70 |
|  |  |  |  |  |
| **Snus use, age groups** |  |  |  |  |
| 16-24 years | 43.1 | 36-50 | 61.0 | 54-68 |
| 25-34 years | 55.7 | 50-62 | 69.2 | 63-75 |
| 35-44 years | 63.6 | 45-80 | 69.7 | 51-84 |
|  |  |  |  |  |
| **Snus use, groups of parity** | |  |  |  |
| No previous child | 54.0 | 49-59 | 70.1 | 65-75 |
| One previous child | 49.6 | 41-59 | 58.9 | 50-67 |
| Two or more previous children | 32.4 | 18-50 | 54.1 | 37-71 |
|  |  |  |  |  |
| **Snus use, educational groups** | |  |  |  |
| Primary/lower secondary | 30.0 | 17-47 | 55.0 | 38-71 |
| Upper secondary | 41.3 | 35-48 | 55.9 | 49-62 |
| Higher education | 71.0 | 64-77 | 81.7 | 75-87 |
|  |  |  |  |  |
| **Smoking, all**  (N=1906 before pregnancy) | 44.9 | 43-47 | 57.7 | 55-60 |
|  |  |  |  |  |
| **Smoking, age groups** |  |  |  |  |
| 16-24 years | 33.5 | 29-38 | 51.5 | 47-56 |
| 25-34 years | 49.0 | 46-52 | 60.4 | 57-63 |
| 35-44 years | 50.6 | 44-57 | 58.6 | 52-65 |
|  |  |  |  |  |
| **Smoking, groups of parity** | |  |  |  |
| No previous child | 50.4 | 47-54 | 65.0 | 62-68 |
| One previous child | 42.7 | 39-47 | 55.5 | 51-60 |
| Two or more previous children | 35.1 | 30-40 | 43.6 | 39-49 |
|  |  |  |  |  |
| **Smoking, educational groups** | |  |  |  |
| Primary/lower secondary | 27.5 | 22-33 | 42.9 | 37-49 |
| Upper secondary | 39.1 | 36-42 | 53.2 | 50-56 |
| Higher education | 72.2 | 68-76 | 80.9 | 77-84 |
|  |  |  |  |  |
| **Dual use, all ****  (N=132 before pregnancy) | 59.1 | 50-68 | 81,1 | 73-87 |

* Tobacco use: daily and occasional use combined

**The quit rates for dual use include those who quit both products, as well as those who quit only one of the products. See text description.
