# Supplementary material for: Trends in maternal use of snus and smoking tobacco in pregnancy. A register study in southern Norway
Source: BMC Pregnancy Childbirth. 2019 Dec 16;19:500. doi: 10.1186/s12884-019-2624-9 (PMC6915947; doi:10.1186/s12884-019-2624-9)
Supplement: Supplementary file 3 — Additional file 3. Stratification of birth weight among snus users, smokers and non-tobacco users. [file 12884_2019_2624_MOESM3_ESM.docx]

**Additional file 3. Stratification of birth weight among snus users, smokers and no tobacco users.**

In our study population we found significantly lower average birthweight in snus users (106 g) and smokers (246 g) than in no-tobacco users. The differences may also reflect differences in gestational age between the tobacco users and non-users. Unfortunately, we had no access to this variable in our dataset. This supplement stratifies birth weight into the same groups as were used in Table 1 and 2 in the main manuscript, as well as in the Additional files Table A1 and A2. Here, all women in the age 16-44 years with records of snus use or cigarette smoking in the third trimester were included in the calculations, not only those with complete records of both snus use and smoking at all three time points. Newborns with birth weight below 500 g were not included in the analyses (unviable births).

**Snus use**

Figure 1 and Table 1 below show lower average birthweight among the youngest snus users (16-24 years, N=78) compared to the non-tobacco users. A smaller, non-significant reduction was found among those aged 25-34 years (N=108) and no difference at all was found among those aged 35-44 years (N=15). We found no comparable age differences in birthweight in the study population as a whole or among the non-tobacco users. This may indicate that the youngest women were more sensitive to birth weight reduction due to snus use. The snus users in 3rd trimester were mainly in the age 16-34 years, reflecting the pattern of snus use before pregnancy in Table 1 and Table S1.

No reduced birth weight was found, compared to the non-tobacco users, in any of the parity-groups of snus users. Snus users with parity 0 had lower birthweight than those with parity 1 (borderline significance), see Table 1 and Figure 2.

No reduced birth weight was found among the snus users with primary/ lower secondary education or among those with higher education compared to the non-tobacco users (Figure 3), but the number of women were low in both groups. The snus users with upper secondary education (N=130) had lower birth weight in their offspring than the non-tobacco users, Figure 3 and Table 1.

The difference in birth weight between snus users and non-tobacco users in our study population seem mainly to be influenced by the snus users in the youngest age group and by those with upper secondary education and no previous child.

**Smoking**

Among the smokers, all age groups as well as all parity groups had reduced birth weight compared to the non-tobacco users. Correspondingly, all educational groups had lower birth weight among smokers than among non-tobacco users. See Figure 1-3 and Table 1.

**Table 1. Birth weight in groups of age, parity and education 2015-2017**

|  | **All** | | **No tobacco in 3. trim** | | **Snus use i 3.trim** | | **Smoking in 3. trim** | |
| --- | --- | --- | --- | --- | --- | --- | --- | --- |
|  | N | Mean weight (CI) | N | Mean weight (CI) | N | Mean weight (CI) | N | Mean weight (CI) |
| 16-44 years | 10211 | 3505 (3494-3516) | 9213 | 3524 (3513-3536) | 201 | 3418 (3338-3498) | 506 | 3278 (3229-3328) |
|  |  |  |  |  |  |  |  |  |
| 16-24 years | 1329 | 3473 (3442-3503) | 1104 | 3509 (3477-3541) | 78 | 3328 (3213-3444) | 110 | 3293 (3182-3404) |
| 25-34 years | 6901 | 3515 (3502-3528) | 6299 | 3529 (3516-3543) | 108 | 3468 (3350-3587) | 300 | 3307 (3244-3370) |
| 35-44 years | 1981 | 3492 (3465-3518) | 1810 | 3516 (3489-3543) | 15 | 3523 (3235-3810) | 96 | 3173 (3053-3292) |
|  |  |  |  |  |  |  |  |  |
| Parity 0 | 4059 | 3414 (3397-3432) | 3664 | 3431 (3412-3449) | 99 | 3315 (3198-3433) | 186 | 3242 (3157-3326) |
| Parity 1 | 3697 | 3574 (3556-3591) | 3346 | 3592 (3575-3610) | 69 | 3549 (3431-3668) | 175 | 3329 (3248-3411) |
| Parity 2 | 2455 | 3576 (3553-3599) | 2203 | 3576 (3553-3599) | 33 | 3450 (3222-3679) | 145 | 3264 (3171-3358) |
|  |  |  |  |  |  |  |  |  |
| Primary/lower secondary | 765 | 3426 ( 3386-3465) | 603 | 3451 (3408-3495) | 28 | 3547 (3336-3758) | 110 | 3261 (3150-3372) |
| Upper secondary | 3567 | 3518 (3500-3537) | 3136 | 3550 (3530-3569) | 130 | 3391 (3294-3488) | 301 | 3281 (3216-3347) |
| Higher education | 5157 | 3523 (3508-3538) | 4922 | 3528 (3513-3544) | 33 | 3524 (3314-3735) | 61 | 3349 (3230-3469) |

**Figure 1. Birth weight in age groups among non-users and users of tobacco**

**Figure 2. Birth weight in parity groups among non-users and users of tobacco**

**Figure 3. Birth weight in educational groups among non-users and users of tobacco**
